# Interaction between Transactivation Domain of p53 and Middle Part of TBP-Like Protein (TLP) Is Involved in TLP-Stimulated and p53-Activated Transcription from the *p21* Upstream Promoter

**DOI:** 10.1371/journal.pone.0090190

**Published:** 2014-03-03

**Authors:** Ryo Maeda, Hidefumi Suzuki, Yuta Tanaka, Taka-aki Tamura

**Affiliations:** Department of Biology, Graduate School of Science, Chiba University, Chiba, Japan; Georgia Regents University, United States of America

## Abstract

TBP-like protein (TLP) is involved in transcriptional activation of an upstream promoter of the human *p21* gene. TLP binds to p53 and facilitates p53-activated transcription from the upstream promoter. In this study, we clarified that *in vitro* affinity between TLP and p53 is about one-third of that between TBP and p53. Extensive mutation analyses revealed that the TLP-stimulated function resides in transcription activating domain 1 (TAD1) in the N-terminus of p53. Among the mutants, #22.23, which has two amino acid substitutions in TAD1, exhibited a typical mutant phenotype. Moreover, #22.23 exhibited the strongest mutant phenotype for TLP-binding ability. It is thus thought that TLP-stimulated and p53-dependent transcriptional activation is involved in TAD1 binding of TLP. #22.23 had a decreased transcriptional activation function, especially for the upstream promoter of the endogenous *p21* gene, compared with wild-type p53. This mutant did not facilitate p53-dependent growth repression and etoposide-mediated cell-death as wild-type p53 does. Moreover, mutation analysis revealed that middle part of TLP, which is requited for p53 binding, is involved in TLP-stimulated and p53-dependent promoter activation and cell growth repression. These results suggest that activation of the *p21* upstream promoter is mediated by interaction between specific regions of TLP and p53.

## Introduction

p53 is one of the most typical tumor suppressors that works as a transcriptional regulator for many genes related to apoptosis induction, DNA repair and cell-cycle repression [1]–[3]. p53 is destabilized by association with MDM2 ubiquitin ligase, which brings p53 to a proteasome-directed proteolytic pathway. When a genotoxin signal enters a cell, intracellular kinase cascades involving ATM/ATR and Chk1/Chk2 functions to phosphorylate p53, which results in release of MDM2 from p53 [4]–[6], and the phosphorylated p53 proteins form a homotetramer and bind to its target sequence of a responding gene [1], [7], [8]. p53 forms a gene family together with TAp63 and p73, all of which have the same consensus sequence [9]–[12].

*p21* (*p21^Waf1/Cip1^*) is a representative p53-responsive gene and antagonizes a Cdk that functions as a cell-cycle engine [13], [14]. p21 mainly works in a G_1_-to-S transition period and triggers G_1_ arrest followed by apoptosis. Hence, p21 is regarded as a potent checkpoint factor and tumor suppressor. Expression of the human *p21* gene is regulated by multiple regulatory factors such as p53, Sp1 and MyoD [15], [16]. The human *p21* gene has two major promoters: a TATA-box-containing downstream promoter and a TATA-less upstream promoter [17], [18]. Since both promoters have p53-binding sites, they are stimulated by genotoxic stresses.

We have identified TLP (TBP-like protein) as a novel regulatory factor for the upstream promoter [19]. TBP (TATA-binding protein) is one of the general transcription factors that binds to a TATA-box promoter element of RNA polymerase II-driven genes [20]. Transcription factor IID (TFIID), which consists of TBP and multiple TBP-associated factors, is recruited to a TATA-containing promoter and triggers transcription initiation [21], [22]. TBP comprises a gene family that includes (TBP-related factor 1) TRF1, TLP/TRF2, TRF3, and TRF4 in addition to TBP [23]–[28]. TLP has 38% identity to the C-terminal conserved region of TBP and binds to transcription factor IIA (TFIIA) more strongly than TBP does [29], [30]. Previously, we demonstrated that TLP inhibits cell growth and induces apoptosis of chicken [31] and mammalian cells [19]. Although TLP has no obvious sequence-specific DNA-binding activity, accumulating evidence indicates that TLP has transcription activation capacity [32], [33]. TLP regulates many genes including *cyclin G2*, *TAp63*, *wee1*, *PCNA*, and *NF1* in addition to *p21* [31], [34]–[37], all of which are categorized as genes involved in cell-cycle regulation, apoptosis induction, tumor suppression and DNA repair. Previously, we clarified that TLP participates in genotoxin-induced and TAp63-mediated apoptosis, and we presented a novel mechanism of *p21* gene regulation involving TLP and p53 [19], [34]. These findings imply that TLP works generally for cell integrity and growth control.

We have demonstrated that TLP activates several TATA-less promoters but not TATA-containing promoters [19]. Other research groups have reported the same phenomenon [37]. We showed that activity of the *p21* upstream promoter is preferentially enhanced by TLP. Moreover, this activation absolutely depends on p53 function, since TLP does not work in promoters carrying mutated p53-responsive elements or in p53-deficient cells. Genotoxin treatment induced nuclear localization of TLP as well as p53, and both factors are co-recruited to the upstream promoter. Furthermore, we obtained evidence of an interaction of TLP with p53 and genotoxin-facilitated recruitment of p53 to the upstream promoter [19].

However, it has not been determined whether TLP-binding ability of p53 is responsible for p53-dependent and TLP-stimulated transcriptional activation of the upstream promoter. In this study, we addressed this issue through mutagenesis of p53, and obtained mutants that retain fundamental transcription-activating function but decreased TLP-stimulated ability. Finally, we found that transcription activation domain 1 (TAD1) residing at the N-terminal region of p53 interacts with the middle part of TLP and works for TLP-mediated transcriptional activation.

## Materials and Methods

### Cell culture, drug treatment, DNA transfection and cell counting

Human HCT116, wild type and p53^−/−^ cells, were maintained in Dulbecco's modified MEM with high glucose content (DMEM-high, Sigma-Aldrich) at 37°C in the presence of 10% fetal calf serum and 5% CO_2_. Etoposide dissolved in dimethyl sulfoxide (DMSO) was added to the medium for some experiments. Transfection of nucleic acids was performed by using Lipofectamine and Plus reagent (Invitrogen) according to the manufacturer's recommendation. Numbers of viable cells were counted by a conventional dye-exclusion method using trypan blue.

### Plasmids

#### Plasmids used in mammalian cells and mutagenesis

FH-TLP, which is the same as pCIneo-FH-TLP described in a previous report [30], is a mouse TLP expression plasmid harboring FLAG and oligohistidine (FH) tags at the N-terminus of TLP. Mouse and human TLPs have identical amino acid sequences. A p53 expression plasmid, pcDNA-FLAG-p53, supplied by Addgene (Cambridge, MA) was modified to pcDNA-HA-p53 (referred to as HA-p53 in this study), which contains an HA tag at the N-terminus. Mutant p53-expressing plasmids were constructed by substitution of one or two amino acid (AA) residues of p53 in pcDNA-FLAG-p53 and pcDNA-HA-p53 plasmids using a PrimeSTAR Mutagenesis Basal Kit (Takara). Expression plasmids for mutant TLPs (R86S, F100E and F114E) were also constructed.

#### Reporter plasmids for luciferase assay

Basically, pGL4.10 vector (Promega) for the luciferase reporter assay was used for plasmid construction. A reporter plasmid (p21up/GL4) containing an upstream region of the human *p21* gene encompassing from −2266 to −1875 was described previously [19].

#### Effector and reporter plasmids for mammalian two-hybrid assay

pBIND vector (Promega) as a bait that includes the GAL4 DNA-binding domain and pACT vector (Promega) as a prey that includes the VP16 activation domain were used for plasmid construction. Open reading frames of TLP/mutant and p53/mutant were linked just downstream from the GAL4 DNA-binding domain of pBIND and VP16 activation domain of pACT vector, respectively. pG5-luc vector (Promega) was used as a reporter plasmid with the luciferase reporter gene.

#### Bacterial expression plasmids

pET-3a vector (Novagen) containing an open reading frame of human p53 for production of FH-p53 and pGEX4T-1 (GE Healthcare) containing an open reading frame of human TBP and mouse TLP for production of glutathione S-transferase (GST)-tagged proteins were described previously [19].

### Short interfering RNA (siRNA)

siRNAs were prepared by using a Silencer siRNA Construction kit (Ambion) as described previously [38]. Sequences of siRNA for human TLP were 5′-UAACAGGGCCCAAUGUAAATT (sense) and 5′-UUUACAUUGGGCCCUAUUATT (antisense). A scrambled sequence of a part of human TFIIAαβ containing 5′-UGGCUGACGACUACUGCGCTT (sense) and 5′-GCGCAGUAGUCGUCAGCCATT (antisense) was used as a control siRNA.

### Luciferase assay

HCT116 p53^−/−^ cells were inoculated into a 24-well plate (1×10^5^ cells/well). Twenty-four hours later, cells were transfected with a reporter plasmid and an effector plasmid and cultured for 24 hr. If necessary, the total amount of transfected DNA was adjusted using pRL-TK (Promega). Cells were harvested and disrupted with Passive Lysis Buffer (Promega). Luciferase activity in lysates was determined using a Dual-Luciferase Reporter Assay System (Promega).

### Bacterially expressed recombinant proteins

The pET series of expression plasmids and pGEX series of expression plasmids were transformed into BL21 and DH5α strains of *E. coli*, respectively. The recombinant proteins were induced by isopropyl 1-thio-β-D-galactoside and purified as described previously [19].

### GST pull-down assay

Purified FH-tagged proteins and glutathione-Sepharose 4B beads (GE Healthcare)-bound GST-tagged proteins were suspended in a binding buffer (50 mM Tris-HCl (pH 7.9), 150 mM NaCl, 1 mM EDTA, 10% glycerol, 0.1% NP-40, and protease inhibitor mixture [30]) and incubated at 4°C for 3 hr. Bound proteins were eluted with SDS sample buffer and detected by immunoblotting as described previously [39].

### Immunoprecipitation of intracellular proteins

HCT116 p53^−/−^ cells transfected with pcDNA-HA-p53/mutants and pCI-neo-FH-TLP were suspended in IP buffer (20 mM Hepes-KOH (pH 7.8), 150 mM NaCl, 1 mM EDTA, 10% glycerol, 0.1% NP-40, and protease inhibitor mixture), disrupted by sonication, and centrifuged at 13,000 rpm for 20 min. The supernatant fractions were collected as whole cell extracts. Protein concentration was determined using a BCA Protein Assay kit (Pierce). Three hundred micrograms of the extract was mixed with anti-FLAG M2 Affinity Gel (Sigma-Aldrich) at 4°C for 3 hr. IgG-Sepharose 6 Fast Flow (GE Healthcare) was used as a control antibody. Bound proteins were eluted with FLAG peptides, boiled for 5 min in SDS sample buffer, and analyzed by immunoblotting.

### Immunoblotting

Proteins were separated by 12.5% SDS-polyacrylamide gel electrophoresis, transferred to an Immobilon-P PVDF membrane (Millipore), and detected by ECL system (GE Healthcare) as described previously [39] by using specific antibodies and appropriate horseradish peroxidase-conjugated secondary antibodies including anti (α)-rabbit IgG and α-mouse IgG. The primary antibodies used included α-p53 antibody (Santa Cruz Biotechnology), α-GST antibody (Ambion), α-glyceraldehyde-3-phosphate dehydrogenase (GAPDH) antibody (Ambion), and antigen-purified α-TLP antibody as described previously [30].

### RT-PCR

Total cellular RNA was prepared by using an RNeasy kit (Qiagen), and reverse transcription-PCR (RT-PCR) was performed as described previously [19]. Briefly, cDNA synthesized from 500 ng of total RNA using Prime Script II (Takara) or avian myeloblastosis virus Reverse Transcriptase XL (Takara) was amplified by PCR using Paq5000 DNA polymerase (Stratagene) and appropriate primer sets. Amplified products were analyzed by 2% agarose gel electrophoresis.

### Statistical analysis

Quantitative data were examined with R Console (ver 3.0.1). Tukey's honestly significant differences test was used to analyze significance of differences between sample means obtained from at least three independent experiments.

## Results

### Affinity of p53 to TLP

**Figure 1:**
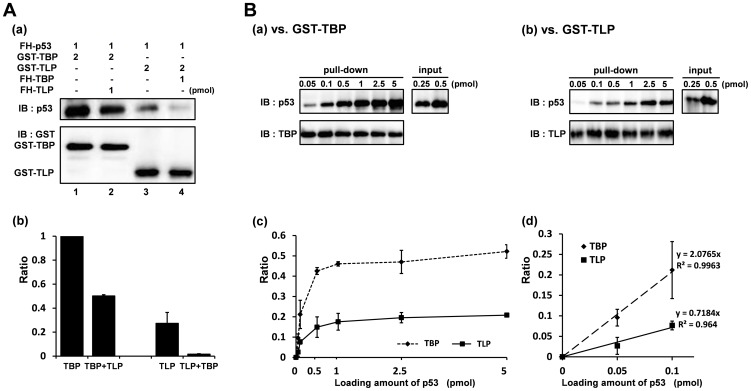
TLP binds to p53 in solution. (**A**) Detection of p53-biding ability of TLP. TLP and TBP were examined for p53 binding by a GST pull-down assay, and affinities of both proteins against p53 were roughly determined by a competitive pull-down assay. (**a**) FH-p53 was challenged to GTS-tagged TBP (lane 1) or TLP (lane 3) as indicated and a simple pull-down assay was performed. TBP/TLP and FH-p53 were detected by α-GST antibody and α-53 antibody, respectively. No signal was detected when only GST tag was used (data not shown). FH-TLP (lane 2) and FH-TBP (lane 4) were co-applied to the GST-fused protein-adsorbed beads together with FH-p53, respectively, as competitors for GST proteins. (**b**) Relative band intensities of lane 2 (TBP+TLP), lane 3 (TLP) and lane 4 (TLP+TBP) to that of lane 1 (TBP) of panel (a) are displayed. (**B**) Comparison of p53-binding affinities of TLP and TBP. GST pull-down assays of lane 1 and lane 2 of panel A-a were performed with increasing amounts of GST-TBP (a) and GST-TLP (b), respectively. input: input protein corresponding to experimental (pull-down) materials. (**c**) Relative band intensity for p53 protein of panel (a). Results of 0.05 and 0.1 pmole of GST proteins of panel (a) are shown again in the magnified graph.

In a previous study, we found that TLP binds to p53 as does TBP even though the AA identity between TLP and TBP is not so high [19], [23]. In this study, we compared the p53-binding capacities of these two proteins by GST pull-down assay. A positive control experiment with a GST-TBP showed strong binding to p53 (Fig. 1A, lane 1). GST alone did not yield any p53 signals (data not shown). The pull-down assay indicated that GST-TLP also binds to p53, though recovered p53 was less than that binding to TBP (Fig. 1A, lane 3). We performed a competition pull-down assay using FH-TBP and FH-TLP as competitors. When FH-TLP was added to the binding reaction of GST-TBP vs. FH-p53, recovered p53 was decreased by 50% of that of the control experiment (Fig. 1A-a and b: lane 2). On the other hand, when TBP was added to the GST-TLP:FH-p53 binding reaction, recovered p53 remained at only 6% of that of the control experiment (Fig. 1A-a and b: lane 4). Hence, it was demonstrated that affinity of TLP to p53 is lower than that of TBP. We compared the p53-binding degrees of the two proteins using increasing amounts of p53. In a control experiment with GST-TBP, the ratio of bound p53 to input fraction reached a plateau at 0.5 (Fig. 1B-a and c), whereas the ratio reached a plateau at 0.2 for GST-TLP (Fig. 1B-b and c). When we focused on data with limited amounts of input p53 substrate (0.05 and 0.1 pmole), degree of the slope of a curve for TLP was about 0.3 to that of TBP (Fig. 1B-d). From these results, TLP-p53 affinity was estimated to be one-third.

### Construction of p53 mutants and their functions in transcription

**Figure 2:**
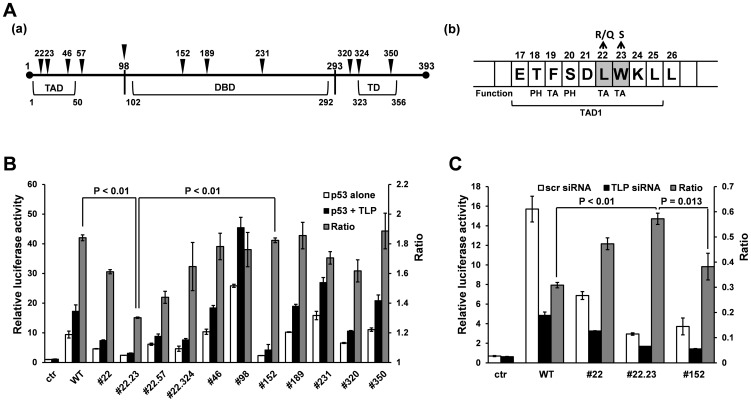
Function of p53 mutants in TLP-stimulated transcriptional activation. (**A**) Schematic representation of the structure of human p53. (**a**) Positions of TAD (transactivation domain), DBD (DNA-binding domain) and TD (tetramerization domain) are indicated with AA positions. Positions of mutation in the examined mutants are shown by vertical triangles. (**b**) AA residues of TAD1 in the TAD region. 22L and 23W have been reported to be critical for transactivation (TA), and 18T and 20S are phosphorylated (PH) amino acids (6). (**B**) Analysis of TLP-stimulated function for individual p53 mutants by an overexpression experiment. Cells were co-transfected with *p21* upstream promoter-carrying reporter plasmid and expression plasmid for p53/mutant alone or p53/mutant+TLP. Results are shown as relative luciferase activities (RLA). Ratio represents RLA of p53/mutant expression to RLA of p53/mutant+TLP expression. Some data were examined by statistical analysis. Since the control experiment (ctr) was performed with a vacant effector plasmid, ratios could not be obtained because measured faint luciferase activities are meaningless. (**C**) Analysis of TLP-stimulated function of representative p53 mutants by a knockdown experiment. TLP siRNA and scrambled (control) siRNA were used as depicted in the figure, and promoter activity was determined as described in panel B.

It has been found that an upstream promoter of the human *p21* gene is potentiated by TLP as is p53 and that TLP stimulates p53-enhanced transcription [19]. We prepared various kinds of mutant p53 and performed a luciferase reporter assay to identify the region required for TLP-stimulated function (*i.e.*, function of TLP that potentiates the ability of p53). Native p53 activated the promoter function by about 10 fold and TLP stimulated p53-enhanced transcription further by 1.9 fold (Fig. 2B, WT). It is well known that the function of p53 strongly depends on its DNA-binding domain (DBD) (Fig. 2A). Although some mutants of DBD were almost inert for basal activation function and we could not determine the TLP-stimulated degree, three mutants, #152, #189 and #231, exhibited significant transcription activation activity. These mutants showed the original degree of TLP-stimulated function (1.6 fold to 1.9 fold), even though a severe mutant, #152, still exhibited a high stimulation index (1.9 fold). These facts suggest that DBD is not responsible for TLP-stimulated function. Results of analysis of the C-terminal TD (tetramerization domain) region (*e.g.*, #320 and #350) also led to the same conclusion.

In the case of a region around the N-terminal *trans*-activation domain (TAD), single AA substitution mutants including #22, #46 and #22.324 exhibited no apparent mutant phenotype for the TLP-stimulated function (Fig. 2B). However, two double-mutants for this region, #22.23 and 22.57, showed relatively low TLP-stimulated functions of 1.3 fold and 1.4 fold, respectively (Fig. 2B). The double-mutant #22.23, in which substituted AA resides in the TAD1 region in the TAD, was the most severe mutant examined. Results are summarized in Table 1. In order to confirm the above results, we conducted a knockdown assay for TLP by using siRNA and representative p53 mutants. As seen in Fig. 2C, TLP siRNA weakened the TLP-stimulated function of native p53 and #152 considerably (30% and 38%, respectively) and that of #22 moderately (48%). We found that #22.23 exhibits the lowest siRNA sensitivity (58%) among the mutants examined, indicating that conclusions obtained from both over-expression and knock-down experiments are consistent. Although differences in the stimulation degrees were not so great in our assays, the results are considered to be highly reproducible and significant from statistical analyses. Consequently, #22.23 was found to be a typical mutant for TLP-stimulated function in p53-directed transcriptional activation.

**Table 1:** Summary of the mutation analysis.

		degree of function*		
name of mutants	details of mutation	BTA	TLP-SF	TLP-BA
p53	wild type	+++	+++	++
#22	L22R	++	++	+
#22.23	L22Q,W23S	+	±	±
#22.57	L22R,D57A	++	+	+
#22.324	L22R,D324Y	++	++	+
#46	S46P	+++	+++	++
#98	P98L	+++	+++	NT
#152	P152L	+	+++	++
#189	A189V	+++	+++	NT
#231	T231I	+++	++	NT
#320	K320N	+++	++	++
#350	L350P	+++	+++	++

* Activation of the mutants are displayed in multiple degrees such as +++ (very strong) ∼ ± (weak).

BTA: basal transactivation function.

TLP-SF: TLP-stimulated function examined by over-expression assay.

TLP-BA: TLP-binding activity.

NT: not tested.

### TLP-binding ability of mutant p53 proteins

**Figure 3:**
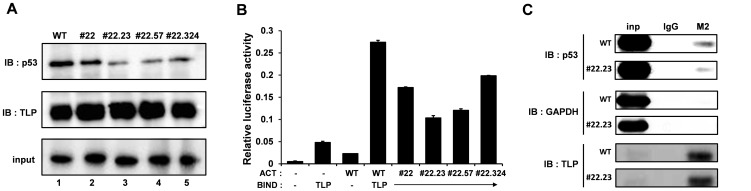
TLP-binding ability of p53 mutants. (**A**) *In vitro* binding of various p53 mutants. A GST pull-down assay was performed as described in the legend of Fig. 1 by using several representative p53 mutants. (**B**) Binding between TLP and p53 or its mutants in cells was examined by a mammalian two-hybrid assay. Binding was monitored by luciferase reporter assay. Plasmids for TLP-containing bait (BIND) and p53/mutant-containing prey (ACT) were introduced into cells as indicated. Since TLP is a transcriptional activator with poor DNA-binding capacity, experiments with bait alone brought significant luciferase activity. (**C**) Immunoprecipitation to detect *in vivo* binding of TLP and p53. FH-TLP and HA-tagged p53 or its mutant (#22.23) were overexpressed in cells and immunoprecipitataied with M2 beads. Immunoprecipitates were analyzed for TLP-associating p53, TLP and GAPDH.

We further investigated *in vitro* TLP-binding ability of several mutants. A GST pull-down assay revealed that #22 and #22.324 had a weakened but still substantial TLP-binding ability (Fig. 3A). On the other hand, TLP-binding abilities of #22.23 and #22.57 were further decreased compared with those of #22 and 322.324 (Fig. 3A). Next, we conducted a mammalian two-hybrid assay to examine an intracellular binding of TLP and p53 mutants. As can be seen in Fig. 3B, #22 and #22.324 showed weaker interaction than wild-type p53, whereas #22.57 and #22.23 showed much weaker interaction. In conclusion, #22.23 is the most typical mutant in both binding assays (Fig. 3A and B). An immunoprecipitation experiment revealed that #22.23 forms fewer intracellular complexes with TLP, suggesting that #22.23 has a weaker TLP-binding affinity than the wilt type in a physiological condition. Since orders of TLP-stimulated function and TLP-binding ability roughly coincided for those mutants, it is thought that the TLP-stimulated property of p53 depends on its TLP-binding ability participating with the TAD1 region.

### Effect of TLP-binding ability of p53 on promoter strength of endogenous *p21* gene

**Figure 4:**
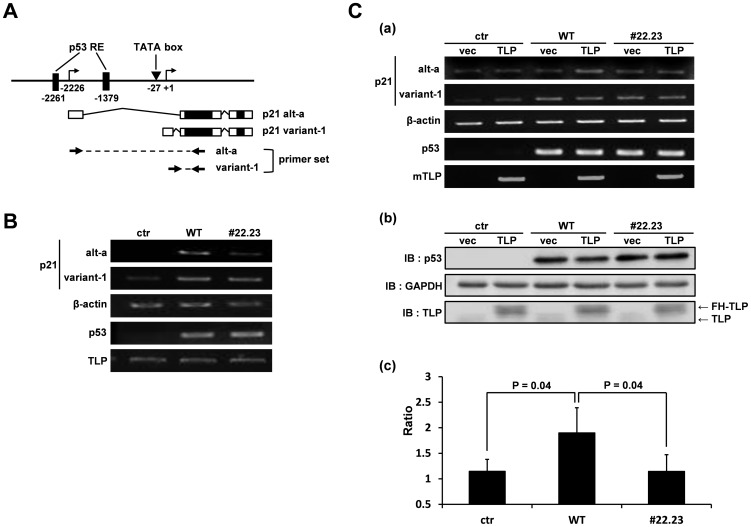
Effect of #22.23 mutation on gene expression from endogenous *p21* promoters. (**A**) Two kinds of major *p21* transcripts produced from the human *p21* gene. Position of exons of *p21* alt-a and *p21* variant-1 transcripts and genomic DNA around the two *p21* promoters are schematically illustrated. Open and solid boxes represent non-coding and coding exons, respectively. Two primer sets indicated by thick arrows were used for RT-PCR to detect variant-1 and alt-a, respectively. (**B**) p53^−/−^ cells were transfected with expression vectors for wild-type and mutant (#22.23) p53, and two species of *p21* transcripts were determined by RT-PCR. Vector: vacant vector. RNAs of endogenous β-actin, p53 and TLP were also analyzed. (**C**) Assays for TLP-stimulated function of wild-type p53 and #22.23. (**a**) Experiments were performed as described in panel B. Cells were transfected with a TLP expression plasmid in addition to a p53 expression plasmid as indicated. ctr and vec: corresponding vacant vectors. (**b**) Amounts of intracellular p53 and #22.23 proteins were also detected by immunoblotting in addition to GAPDH and endogenous and exogenous TLPs. (**c**) Degree of increase in alt-a transcripts stimulated by exogenous TLP in p53-expressing cells. Ratios of band intensities of alt-a of panel (a) in vacant vector-introduced cells to that in TLP overexpressed cells were calculated for three kinds of cells.

In a previous study, we found that the *p21* upstream promoter is greatly dependent on TLP compared with the downstream promoter [19]. The upstream and downstream promoters mainly produce alt-a and variant-1 transcripts, respectively. We exogenously expressed native p53 or #22.23 and detected endogenous *p21* transcripts by RT-PCR (Fig. 4A). Compared with wild-type p53-expressing cells, the amount of alt-a was significantly small in #22.23-expressing cells, whereas that of variant-1 decreased only slightly (Fig. 4B). These results indicate that the upstream promoter is more sensitive to the #22.23 mutation than is the downstream promoter even though both promoters need p53 function for substantial levels of transcription. Next, we investigated effects of exogenously expressed TLP on p53-enhanced transcription for the two kinds of transcripts. TLP increased production of alt-a but not that of variant-1 when wild-type p53 was co-expressed. In contrast, the #22.23 mutant did not bring a stimulation effect on alt-a expression (Fig. 4C-a and c). Taken together, the results indicated that TLP-binding function of p53 is specifically exhibited in the upstream promoter.

### TLP-binding ability of p53 and TLP-mediated cell death

**Figure 5:**
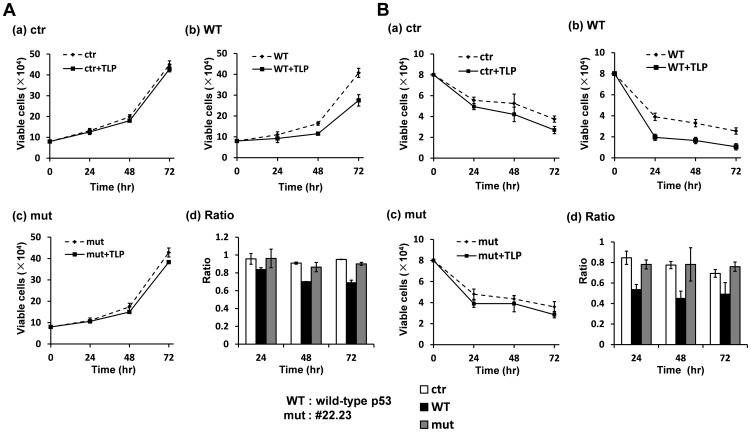
Effect of #22.23 mutation on cell growth and etoposide-induced cell death. (**A**) Five-hundred thousand p53^−/−^ cells in a dish were cultured for 24 hr. Cells were transfected with an expression plasmid for p53 (WT) or #22.23 (mut) together with a TLP expression plasmid. After 24 hr, 8×104 cells were replated and maintained. Cell numbers were counted every 24 hr (panels a–c). ctr: vacant plasmid. (**d**) Cell numbers at each time shown in panels a–c are displayed as ratios to the initial cell number. (**B**) Experiments were performed as described above, but replated cells were maintained in a medium containing 30 µM etoposide to examine the effect of TLP on apoptotic cell death (**a–c**). Numbers of remaining viable cells were counted. (**d**) Data are summarized as described above.

Cells expressing a substantial level of p21 proteins undergo growth arrest and occasional cell death. First, p53^−/−^ cells were transfected with various kinds of expression plasmids and cell numbers were scored every 24 hr. Compared with vacant plasmid-introduced cells (Fig. 5A-a, ctr), TLP overexpression exhibited considerable growth inhibitory effect in exogenously p53-expressing cells (b: WT), whereas this effect was not prominent in #22.23-expressing cells (c: mut). Results are summarized in panel d (Fig. 5A). Next, we investigated effect of TLP on apoptosis. Cells were treated with etoposide to induce cell death. In the case of vacant plasmid-introduced cells, cells died gradually (Fig. 5B-a, ctr), whereas cells died slightly faster with a cell death-facilitating rate (CDFR) of 0.7–0.85 when TLP was over-expressed (Fig. 5B-a, ctr+TLP). CDFR of TLP (0.45–53) was much greater than that in the control experiment in wild-type p53-expressing cells (Fig. 5B-b). On the other hand, CDFR of TLP in #22.23-expressing cells (0.73–0.77) was almost the same as that in the control experiment (Fig. 5B-c). Results are summarized in panel d (Fig. 5B). The results of these experiments suggest that obtained phenomena are exhibited via interaction of TLP and p53 and might be involved in facilitated expression of *p21* gene.

### Examination using mutant TLPs

**Figure 6:**
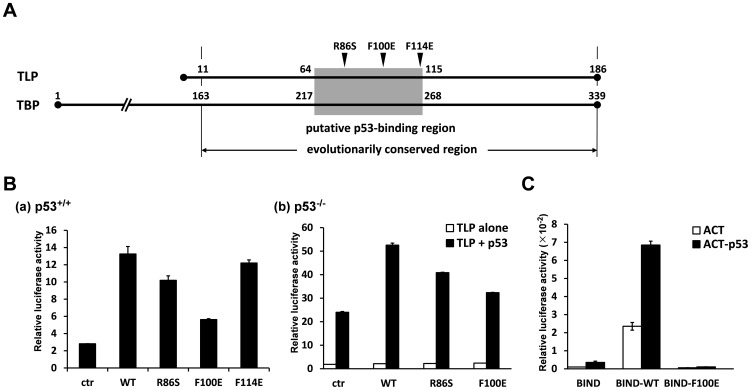
Examination of mutant TLPs on transcriptional activation and p53 binding. (**A**) Structural relationship between TBP and TLP. Amino acid numbers are indicated from N-termini. TLP covers the evolutionally conserved region of TBP. A putative p53-binding region in TBP deduced from deletion analyses [44] and its TLP counterpart (from 63 to 115) are depicted as a gray area. Positions of AAs of the TLP mutants used in this study (R86S, F100E, and F114E) are indicated with vertical arrowheads. (**B**) Transcription activation function of wild-type (WT) and mutant TLPs were assayed in native (**a**) and p53^−/−^ (**b**) cells. (**C**) Binding of TLP and p53. Wild-type and F100E TLPs were analyzed for the p53-bidnding ability by two-hybrid assay.

**Figure 7:**
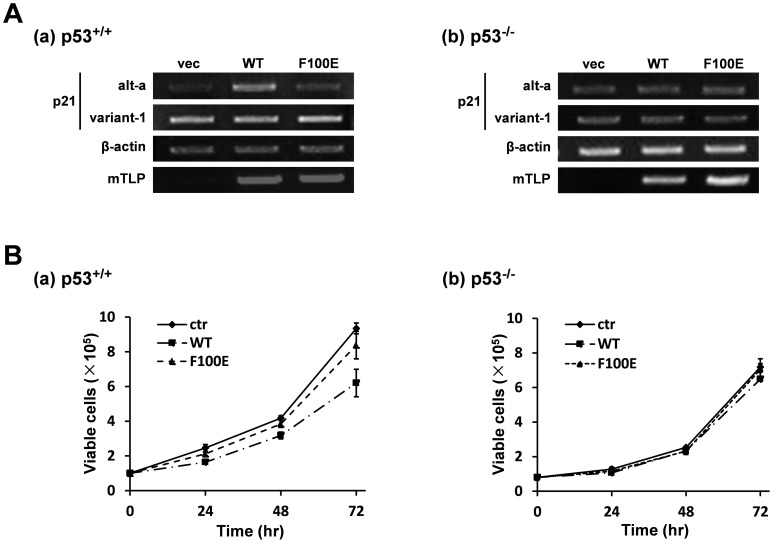
Effect of F100E mutation of TLP on the expression of endogenous *p21* gene and cell growth. (**A**) Wild-type (a) and p53^−/−^ cells (b) were transfected with expression vectors of wild-type and mutant (F100E) TLPs, and two species of *p21* transcripts were determined by RT-PCR as described in a legend of Fig. 4. (**B**) Wild-type and mutant TLP-transfected native (**a**) and p53^−/−^ (**b**) cells were cultured for 24 hr. Cells (1×10^5^) were replated and cell numbers were counted every 24 hr. ctr: vacant plasmid.

We further confirmed the p53-TLP interaction on p53-mediated transcriptional activation by using mutant TLPs that have impaired p53-binding ability. We prepared three kinds of mutant TLPs; R86S, F100E, and F114E. Positions of these mutated AAs are located in a region of TLP, whose corresponding AAs are critical for transcriptional activation function of TBP and the binding to TFIIA (*i.e.*, another general transcription factor), and are included in a putative p53-binding region of TBP (Fig. 6A) [30], [40], [45]. Through a function assay, we found that R86S and F100E exhibit weak and strong mutant phenotypes in transcription activation function, respectively, in a p53-dependent manner (Fig. 6B). Moreover, F100E was found to lose its p53-binding ability (Fig. 6C). These results suggest that TLP binds to p53 via its middle region. Overexpressing experiments demonstrated that 100^th^ Phe (F100) of TLP is required for stimulation of alt-a but not variant-1 *p21* transcripts (Fig. 7A-a). This stimulation occurred in a p53-dependent manner, because amounts of alt-a were similar in WT- and F100E-transfected p53^−/−^ cells (Fig. 7A-b). Furthermore, growth repression of wild-type cells was observed for WT-transfected cells but not for F100E-transfected cells (Fig. 7B-a), and this repression disappeared when p53-negative cells were used (7B-b). Finally, we concluded that substantial transactivating function of p53 to the *p21* upstream promoter and subsequent growth repression needs the binding of TAD1 domain of p53 to the middle region of TLP.

## Discussion

p53 is one of the most popular cellular regulators in vertebrates. Upon genotoxic stresses, p53 is phosphorylated and dissociated from MDM2 ubiquitin ligase, which destabilizes p53 [5], [6]. Stabilized and nucleus-translocating p53 binds to a specific DNA sequence as a homotetramer and regulates expression of genes related to growth repression, apoptosis induction, stress response, checkpoint and DNA repair [2], [3]. Since p53 is such a wide-range cellular regulator, various proteins can bind to p53 to modify its function, dynamics and stability [41]. Some transcription-relating factors such as general transcription factors (*e.g.*, TFIID, TBP and TFIIH) and transcriptional co-activators (*e.g.*, p300, P/CAF) bind to p53 [42]–[46]. Previously, we demonstrated that TLP is a novel p53-binding protein [19]. In this study, we examined the TLP-binding property of p53 in detail. From competitive and semi-kinetic GST pull-down assays, we estimated that the binding strength of p53 to TLP is about one-third of that to TBP. This estimation seems plausible since TLP is only 38% identical to a C-terminal conserved region that serves as a protein-binding surface of TBP.

Through an extensive mutant analysis, we found a TLP-binding region of p53. The #22.23 mutation, in which AA substitutions reside in TAD1, exhibited the greatest defect in TLP-binding ability among the mutants examined. Since #22.23 exhibited a considerable defect in both *in vitro* and *in vivo* binding assays, L22 and W23 are thought to be critical for the binding. We concluded that TLP binds to the N-terminal TAD1 region of p53. In two mutated AAs in #22.23, W23 may be much critical, since #22 and #22.324 are not obvious mutants for TLP binding. Alternatively, L22R may be a partial mutation and W23S may strengthen the mutation phenotype.

p53 contains multiple functional domains including N-terminal TAD, central DBD and C-terminal TD, all of which contribute to transcriptional activation function in each way [47]. In order to identify the region of p53 responsible for the TLP-stimulated function in p53-activated transcription from the *p21* upstream promoter, we performed promoter assays through overexpression of various types of p53 mutants together with TLP. #320 and #152, which have AA substitutions in TD and DBD respectively, exhibited lower transcription activation ability. However, these mutants still showed a native TLP-stimulated function. On the other hand, all mutants that have AA substitutions in TAD1 exhibited decreased function compared with that of the wild type. Among the mutants, #22.23 was the most severe and exhibited the lowest TLP-binding capacity. Moreover, orders of the mutant phenotypes in the function assay and binding assay were basically consistent. Consequently, we concluded that TLP-stimulated function of p53 depends on its TLP-binding ability participating with the TAD1 region. Since T18 and S20 are phospholylated upon genotoxic stress (Fig. 2A-b), we constructed T18K and S20P mutants and examined their functions. However, since they exhibited native functions (data not shown), phospholyration of TAD1 may not be needed for TLP binding.

Through mutation analyses, we identified a p53-bindiong region of TLP (Fig. 6B and C). This is the first report to specify p53-binding AA residues for the TBP-family proteins. Like p53 mutants for TLP binding, the typical mutant TLP (F100E) exhibited lower functions for p53-dependent transcriptional activation from the *p21* upstream promoter and cell growth repression in addition to p53-binding. Consequently, we were able to conclude that TLP-mediated p53 function needs direct interaction of specific regions of these two proteins (*i.e.*, the TAD1 of p53 and a middle region of TLP around the 100^th^ AA residue). TBP has been shown as one of the typical p53-interactive transcription factors [42]–[44]. Since locations of AAs needed for p53 binding are analogous between TBP and TLP (Fig. 6A), p53-binding fashion may be similar for both proteins.

Unlike TLP, TBP binds to p53 via the C-terminal TD in addition to the TAD [45]. It is notable that our immunoprecipitation assay could detect intracellular TLP-p53 complex (Fig. 3C) but not TBP-p53 (data not shown), even though binding strength between TBP-p53 in solution is greater than that between TLP-p53 (Fig. 1). Moreover, evidence relating to *in vivo* binding of TBP-p53 and p53-dependent transcription activation function of TBP has not yet been obtained. Hence, TBP may not functionally interact with p53, and TLP might be unique among TBP family proteins for functional p53 binding. We assume that TBP-associated factors, but not TBP, in TFIID form a functional complex in cells. Actually, it has been reported that TFIID interacts with the TATA-containing downstream promoter of the *p21* gene, which also contains a p53-binding site.

The significance of TLP-p53 binding is not clear at the present time. TLP may stabilize p53 or facilitate formation of a p53 homotetramer. However, we also assume that TLP directly works in a transcriptional regulation process. We have demonstrated that the TATA-less upstream promoter of the *p21* gene is preferentially stimulated by TLP [19]. It is speculated that the upstream promoter-bound p53 is regulated by unknown factors in addition to TLP. Suzuki *et al.* clarified that the upstream promoter is further stimulated by TFIIA (manuscript in preparation). It is generally known that TLP binds to TFIIA more strongly than does TBP [29], [30]. Taken together, our results suggest that the weak p53-binding activity of TLP is augmented by TLP-associating TFIIA in the upstream promoter. This may be a reason why TLP can exhibit its function in the upstream promoter even though its p53-binding affinity is low. Generally, a transcriptional activation domain serves as a binding surface to basal transcription machinery. Hence, TLP might bind to TAD1 and mediate a transcriptional activation signal of p53 to the basal machinery. In other words, TLP might work as a co-activator of p53 in the *p21* upstream promoter.

Reason of the existence of dual promoters of *p21* gene is not elucidated so far. This promoter structure may have an advantage to express *p21* gene in various cellular situations. For example, in contrast to the upstream promoter, *p21* downstream promoter is mainly governed by rather constitutive factor including TBP and TBP-associated factors in addition to p53 [46]. Hence, the downstream promoter may function preferentially in a usual cellular condition. On the other hand, the upstream promoter may be more important in unusual and/or inducible conditions such as stress response, apoptosis induction, and development & differentiation, since function of TLP is needed in such situations [19], [24], [31], [34].
